# Melatonin facilitates extinction, but not acquisition or expression, of conditional cued fear in rats

**DOI:** 10.1186/1471-2202-15-86

**Published:** 2014-07-15

**Authors:** Fulian Huang, Zehua Yang, Xiaoyan Liu, Chang-Qi Li

**Affiliations:** Department of Anatomy and Neurobiology, School of Basic Medical Sciences, Central South University, Tongzipo Road 172, Changsha, Hunan 410013 P.R China; Department of Physiology, Yiyang Medical College, Yingbin Road 516, Yiyang, Hunan 413000 P.R China

**Keywords:** Melatonin, Cued fear, Fear conditioning, Fear extinction, PTSD, Rats

## Abstract

**Background:**

Previous studies have shown that melatonin is involved in the processes that contribute to learning and memory. At present study, we tested the effects of exogenous melatonin (2.5 mg/kg) on the acquisition, expression and extinction of cued fear in rats.

**Results:**

Results showed that a single afternoon administration 30 min before conditioning has no effect on the acquisition of cued fear. Compared to rats injected with vehicle, rats injected with melatonin 30 min before extinction training presented a significant lower freezing during both extinction training and extinction test phases, however, freezing response did not differ for the initial four trials during extinction training. Melatonin injected immediately after extinction training was ineffective on extinction learning.

**Conclusions:**

These results suggest that melatonin, at the dose applied in this study, facilitates the extinction of conditional cued fear without affecting its acquisition or expression, and melatonin facilitates cued fear extinction only when it is present during extinction training. These findings extend previous research on the melatonin effects on learning and memory and suggest that melatonin may serve as an agent for the treatment of anxiety disorders such as posttraumatic stress disorder (PTSD).

## Background

Melatonin is a pineal gland hormone synthesized and secreted at night in vertebrates. Besides its moderator role in the regulation of circadian rhythms and sleep [1–3], melatonin has shown to display antidepressant and anxiolytic properties in animal models [4–6]. Moreover, it is thought to be involved in modulating complex processes such as learning and memory [7] by binding to receptors [MT(1)/MT(2)] which are widely distributed in the brain [8]. For instance, melatonin was shown to possess memory facilitating effects in the novel object recognition task [9, 10] and the olfactory social memory test in rats [11]. Other reports showed that melatonin enhances performance in a verbal association task [12] and improves memory acquisition under stress [13]. And mice lacking melatonin MT2 receptors showed impaired performance tested in an elevated plus-maze [14]. However, there were also the opposite outcomes. It was reported that zebrafish has a better memory performance during the day than during the night and melatonin is necessary for the suppression of memory during the night [15]. Karakas et al. [16] injected locally melatonin to the amygdala and found that such treatment impaired spatial memory performance of the rats. In addition, we have recently demonstrated that melatonin impairs the acquisition of contextual fear memory [17].

To study the involvement of melatonin in memory processing, we tested the role of melatonin in fear conditioning and fear extinction. Pavlovian fear conditioning involves pairing an initially neutral conditioned stimulus (CS) such as a tone or context with an aversive unconditioned stimulus (US) like a footshock. After several pairings of these stimuli, the CS comes to elicit conditional fear responses such as defensive behavioral responses (e.g., freezing). Considerable evidence suggests that the amygdala is an important site of the neural circuits related to both cued and contextual fear conditioning. However, the hippocampus is usually required only for contextual task [18, 19]. After conditioning, repeatedly presentations of the CS in the absence of the US results in a progressive reduction of the conditioned response. This process is called extinction. Numerous studies have suggested that extinction is a form of new, context-dependent learning [20, 21]. These studies suggest that the memory formed during conditioning is not eliminated during extinction but rather is suppressed by extinction learning, which likely attribute to plasticity at distinct synapses from those mediating acquisition. Converging evidence has identified a network of brain structures including the amygdala, prefrontal cortex, and hippocampus [22–25] that supports the acquisition, storage, retrieval, and contextual modulation of fear extinction.

As melatonin has been implicated in the acquisition of contextual fear conditioning [17], it is of particular interest to examine whether melatonin is involved in regulation of some aspect of conditional cued fear. Thus, the present study was designed to assess the effects of systemic injections of melatonin with good penetration through the blood–brain barrier on the acquisition, expression, and extinction of conditional cued fear in rats.

## Methods

### Subjects

Experimental procedures were performed on adult male Sprague–Dawley rats (270–320 g) obtained from the Laboratory Animal Center of Central South University, Changsha, Hunan, China. After arrival, the rats were housed one per cage at 25°C and an appropriate level of humidity, with *ad libitum* access to food and water. A 12:12 light–dark cycle was maintained, with all procedures occurred between 3:00 PM. and 6:00 PM. Prior to all behavioral procedures, the rats were handled daily for 1 week in order to eliminate handling stress as a confounding variable. Experiments were conducted according to the *National Institutes of Health Guide for the Care and Use of Laboratory Animals*, and experimental protocols were approved by the animal care and use committee of Central South University.

### Behavioral apparatus

Rats underwent fear conditioning and fear extinction in two different observation chambers (Huaibei Zhenghua Biological Equipment Co. Ltd., Anhui, China). The chambers (46 cm × 46 cm × 46 cm, without ceiling) were situated within a sound-attenuating cabinet individually. On the ceiling of the cabinet was a speaker to present acoustic CSs and a 8 W white house light to illuminate the chamber. A ventilation fan mounted on the right wall of the cabinet provided a 60 dB background noise and air exchange. For fear conditioning, the walls of the chamber (context A) were made of black opaque Plexiglas. The floor of context A consisted of 23 stainless steel bars (6 mm in diameter) spaced 20 mm apart that were connected to a shock generator and scrambler for the delivery of foot-shock USs. The presentation and sequencing of all stimuli was controlled by a custom written computer program. The chamber was thoroughly cleaned with water and dried between sessions. For extinction training and CS testing, rats were placed in a novel chamber (context B) with transparent Plexiglas walls and a smooth black plastic floor to minimize generalization to the conditioning chamber. The chamber was cleaned with 75% ethanol before each session.

### Cued fear acquisition

**Figure 1:**
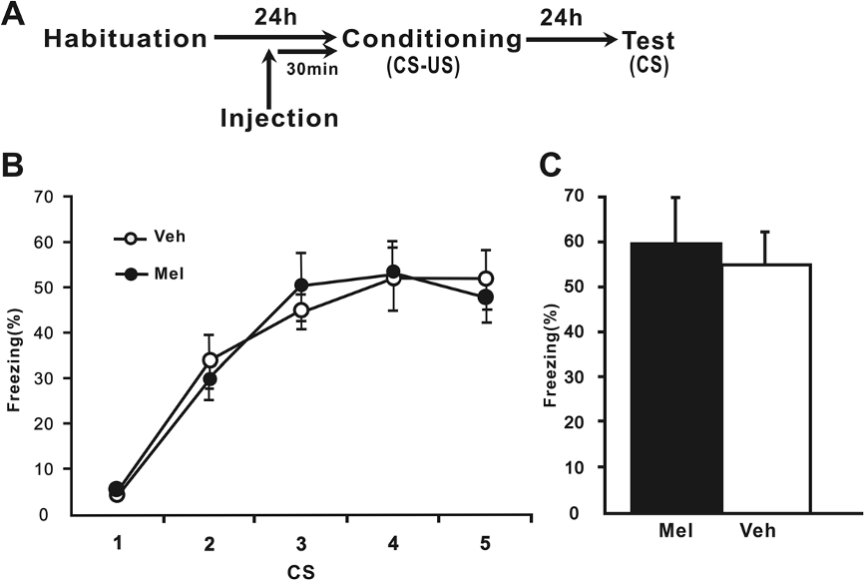
**Melatonin does not affect cued fear acquisition. (A)** Schematic of the behavioral procedure used. Behavior procedure involved three training phases: habituation, fear conditioning and test. Rats were administrated melatonin 30 min before conditioning phase. **(B)** Percent freezing during the five cues was shown for the melatonin group (n = 10) and the vehicle group (n = 10) during conditioning phase. **(C)** Percent freezing to the five cues during test phase. Mel = melatonin group, Veh = vehicle group. All data are represented as mean ± SEM.

Experiment 1 investigating the effects of melatonin on the acquisition of cued fear (Figure 1A) involved three phases: habituation (context A), fear conditioning (context A) and testing (context B), each separated by 24 h to allow for memory consolidation. During habituation phase (Day 0), rats were habituated to the conditioning chamber (context A) for 20 min with no stimuli presented. On day 1 (fear conditioning phase), rats were allowed to explore the chamber for 3 min. At the end of 3 min rats were subjected to five trials of audio tone (CS) and foot shock (US) with an inter-trial interval of 60 sec. Audio tone (4 kHz, 80 dB, 20 sec duration) was followed immediately by a footshock (0.5 mA, 0.5 sec duration) from the metal grid floor. The rats remained in the training box for 60 s following the last CS-US pairing, after which they were returned to the home cages. On Day 2 (testing phase), after a 3 min acclimation, rats received 5 tone alone presentations with an inter-trial interval of 60 sec.

### Cued fear extinction

**Figure 2:**
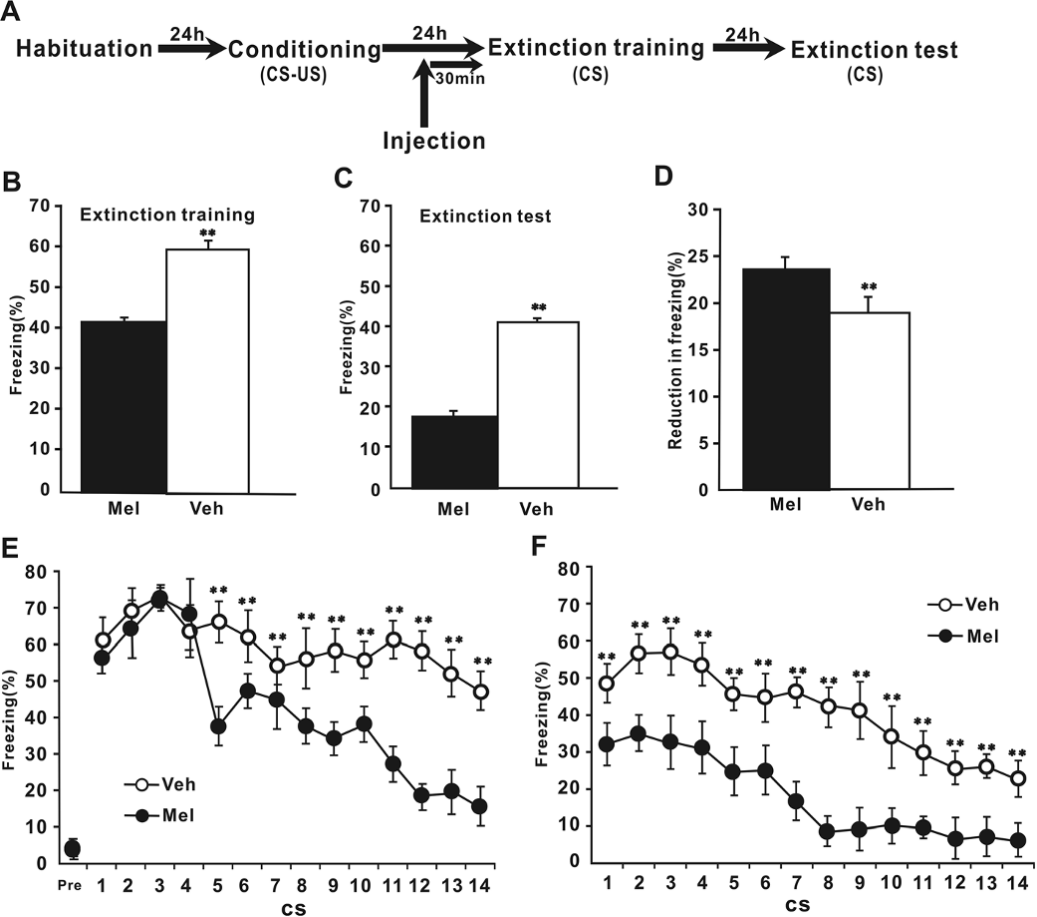
**Melatonin facilitates extinction, but not expression, of cued fear. (A)** Schematic of the behavioral procedure used. Behavior procedure involved four training phases: habituation, fear conditioning, extinction training and extinction test. Rats were administrated melatonin 30 min before extinction training. **(B)** Percent freezing to the CSs, averaging across all trials, was shown for the melatonin group (n = 10) and the vehicle group (n = 10) during extinction training. **(C)** Percent freezing to the CSs, averaging across all trials during extinction testing. **(D)** Reduction in percent freezing between the extinction training and extinction test based on the data in B and C. **(E)** Percent freezing to the CSs during extinction training. Freezing was also scored during a 3 min acclimation period (Pre-CS). **(F)** Percent freezing to the CSs during extinction testing. Data used in **B** and **C** were reanalyzed for the trials of percent freezing. Mel = melatonin group, Veh = vehicle group. ** *P* < 0.01 for comparisons between the melatonin group and the vehicle group. All data are represented as mean ± SEM.

Experiment 2 and Experiment 3 investigating the effects of melatonin on cued fear extinction (Figure 2A) involved four phases: habituation (context A), fear conditioning (context A), extinction training (context B) and extinction test (context B), each separated by 24 h to allow for memory consolidation. In both experiments, cued fear was induced in nondrugged, naı¨ve rats described above. On Day 0 (habituation phase) and Day 1 (fear conditioning phase), the habituation and fear conditioning trainings were same as described in Experiment 1. Rats were matched into two groups that received either melatonin or vehicle based on freezing during the third training CS. Twenty-four hours after fear conditioning (Day 2, extinction training phase), rats were placed in context B and were allowed to acclimate for 3 min. Following this, rats received 14 tone (4 kHz, 80 dB, 20 sec duration) alone presentations with an inter-trial interval of 60 sec. The rats were immediately returned to their home cages 60 s after the last tone presentation. On day 3 (extinction test phase), rats received 14 tone alone presentations in context B as described on day 2.

### Drugs

Melatonin was purchased from Sigma-Aldrich and was tested at doses of 2.5 mg/kg. This compound was dissolved in small volume of 75% ethanol and further diluted in saline (vehicle) to the final volume immediately before administration. The final concentration of alcohol was <0.5%. Rats were administered intraperitoneally (i.p.) in a volume of 5 ml/kg body weight. Vehicle-treated rats received the same volume via i.p. injection.

### Timing of melatonin injection

**Figure 3:**
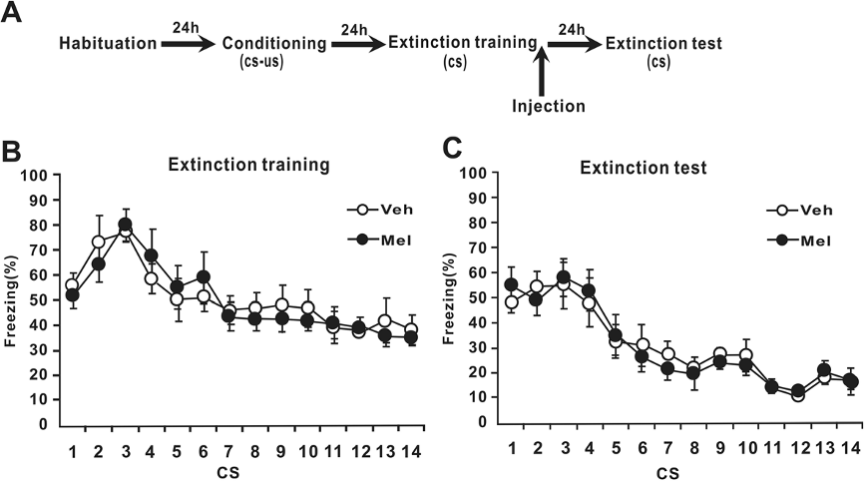
**Melatonin injected immediately after extinction training has no effect on extinction learning. (A)** Schematic of the behavioral procedure used. Behavior procedure involved four training phases: habituation, fear conditioning, extinction training and extinction test. Rats were administrated melatonin immediately after extinction training. **(B)** Percent freezing to the CSs was shown for the melatonin group (n = 6) and the vehicle group (n = 6) during extinction training. **(C)** Percent freezing to the CSs during extinction testing. Mel = melatonin group, Veh = vehicle group. All data are represented as mean ± SEM.

Rats were given i.p. injections of melatonin (2.5 mg/kg) or vehicle 30 min before fear conditioning in Experiment 1 (Figure 1A), 30 min before extinction training in Experiment 2 (Figure 2A) or immediately after extinction training in Experiment 3 (Figure 3A).

### Statistical analyses

Freezing was used as the measure of conditional fear response during fear conditioning phase, extinction training phase and testing phase. Freezing is characterized by cessation of movement except that required for respiration (Blanchard and Blanchard, 1969). The total time spent freezing during every 20-s tone CS was scored offline with a digital stopwatch from digital videos. Observers scoring freezing were blind to the treatments. Freezing is presented as the percent time spent freezing (time spent freezing/total time × 100). Total session means were analyzed with Student’s *t*-test. Multiple trial data were analyzed with two-way repeated-measures analysis of variance (ANOVA). Post-hoc comparisons were performed with the Tukey HSD method. All data were represented as mean ± SEM. Significant level was set at *p* < 0.05. Statistics were run on SPSS (Version 13; SPSS, Chicago, IL).

## Results

### Experiment 1: Effects of melatonin on the acquisition or retention of conditional cued fear

First, we investigated the effects of melatonin on cued fear acquisition. We injected melatonin 30 min before fear conditioning. During the conditioning phase (Figure 1B), the melatonin and vehicle groups presented a progressive increase in the conditioned fear response across trials. A two-way repeated-measures ANOVA of percent freezing found there was a significant effect of trial (*F* (4, 72) = 278.343, *p* < 0.001) but not group (*F* (1, 18) = 0.010, *p* > 0.05) or interaction of group and tone block (*F* (4, 72) = 2.876, *p* > 0.05), indicating that the two groups showed equivalent fear learning. At 24 h post-conditioning, the rats were presented with the CS in a novel context. There was no a significant group effect of percent freezing (*p* > 0.05) (Figure 1C). These data suggest that melatonin does not affect the acquisition and retention of cued fear.

### Experiment 2: Effects of pre-extinction melatonin on extinction and expression of conditional cued fear

Next, we assessed the effects of melatonin on the expression and extinction of cued fear memory. Two groups of naı¨ve rats underwent fear conditioning. One day after training, rats were injected with melatonin or vehicle 30 min before extinction training. The next day, they were subjected to a drug-free extinction test.

During the extinction training phase, a significantly lower freezing was observed in the melatonin group in comparison to the vehicle group (*p* < 0.001) (Figure 2B). During the extinction test phase, level of freezing was significantly lower in both groups when compared with the respective level measured during extinction training (*p <* 0.001) (Figure 2C), indicating successful retrieval of extinction learning. A significant difference in the level of freezing between the two groups was observed during extinction testing (*p <* 0.001) (Figure 2C). Moreover, in the melatonin group, the reduction in the levels of freezing from extinction training to extinction testing was significant compared to the vehicle group (p < 0.001) (Figure 2D). These results suggest that injection of melatonin 30 min before the extinction training facilitates the acquisition and retention of extinction learning.

The data were reanalyzed for the trials of percent freezing using two-way repeated-measures ANOVA. During extinction training, the two groups presented a gradual reduction in freezing across trials (trial, *F* (13, 234) = 83.843, *p* < 0.001) (Figure 2E). The melatonin group showed a significant decrease in the level of freezing compared to the vehicle group (group, *F* (1, 18) = 749.594, *p* < 0.001; group × trial, *F* (13, 234) = 28.065, *P* < 0.001). Post hoc comparisons indicated that rats injected with melatonin or vehicle expressed similar freezing response during the initial four trials (all, *P* > 0.05) (Figure 2E). However, the melatonin group presented a significant lower freezing in the other trials (all, *P* < 0.001) (Figure 2E). In contrast, during extinction test phase, the level of freezing response for the melatonin group was significantly lower at each trial compared with the vehicle group (p < 0.001 for all trials) (Figure 2F). These results suggest that melatonin injection doesn’t affect the expression of conditional fear.

To confirm that confounding effects of nonspecific freezing responses to context itself were not somehow influencing freezing responses, we also recorded the time spent freezing during the 3 min pre-CS period of extinction training on day 3 in context B. There was no significant difference in the level of freezing between the melatonin group and vehicle group (*P* > 0.05) (Figure 2E), suggesting that melatonin does not significantly affect unconditioned freezing responses to the chamber alone.

### Experiment 3: Effects of post-extinction melatonin on extinction of conditional cued fear

We also administered melatonin immediately after extinction training to determine the effects of melatonin on extinction when it is administrated at different time points.

During the extinction training phase (Figure 3B), the level of freezing by rats in the melatonin and vehicle groups decreased across trials (*F* (13, 130) = 46.553, *p* < 0.001). There was no a significant effect of group (*F* (1, 10) = 0.411, *p* > 0.05) and no significant trial-by-group interaction (*F* (13, 130) = 2.254, *p* > 0.05). During the extinction test session (Figure 3C), both groups also presented a progressive decrease in freezing responses across trials (*F* (13, 130) =93.234, *p* < 0.001). There was no a significant effect of group (*F* (1, 10) = 0.049, *p* > 0.05) and no significant trial-by-group interaction (*F* (13, 130) = 1.566, *p* > 0.05). These results suggest that melatonin administration immediately after extinction training has no effect on the extinction of conditional cued fear.

## Discussion

We presented data that suggest a differential effect of melatonin on acquisition and extinction of conditional fear in rats. For cued fear, melatonin (2.5 mg/kg) following a single i.p. administration had no effect on the acquisition and retention of conditional cued fear response in rats (Figure 1). This was indicated by the observation that the melatonin and vehicle groups showed an identical increase of freezing with training trials during the conditioning phase on Day 1 and an identical great conditional fear during drug-free testing phase on Day 2.

On the other hand, pre-extinction injection of melatonin facilitated the acquisition and retention of cued fear extinction (Figure 2). These effects could not be attributed to changes of nonspecific responses to context itself that followed melatonin injection (Figure 2E). In addition, augmented locomotion or anxiolytic property of melatonin may be indirect factors that attenuate freezing behavior. However, our previous study [17] showed that melatonin injection with the same dose as used in the present study did not induce significant difference in spontaneous locomotor activity or anxiety behavior as assessed with the open field test. Thus, facilitation of extinction learning by melatonin could not be attributed to stimulated locomotion or altered anxiety behavior. Further, initial freezing levels during extinction training were similar for melatonin- and vehicle-treated rats (Figure 2E). These results argue that melatonin has no interference with the expression of cued conditional fear, which also confirms further its effect on fear extinction. Collectively, although there were no effects on the acquisition or expression of cued fear, melatonin facilitated cued fear extinction. This finding that melatonin exerts opposing influences on the acquisition and extinction of conditional fear is in accordance with many other studies showing that acquisition and extinction are distinct learning processes. There exists behavioral, systems, and molecular differences between acquisition and extinction [21, 26–28]. Furthermore, in line with the present finding, it has been found that melatonin facilitates the extinction of active avoidance reflex, whereas memory acquisition is not influenced [29].

In contrast with the ability of pre-extinction injection of melatonin to facilitate the extinction, a post-extinction injection of melatonin did not alter the extinction (Figure 3). Therefore, these results suggest that melatonin facilitates cued fear extinction only when it is present during extinction training. This finding is in accordance with a previous study [15]. In this study, using a modified active-avoidance conditioning (AAC) paradigm, melatonin applied before training significantly suppressed long-term memory formation in zebrafish, however, no effect of melatonin on long-term memory formation was observed when it was given directly after training. These observations, together with our present results, suggest that melatonin has a profound influence on memory consolidation at a relatively early stage of long-term memory formation.

We have shown previously that melatonin impairs the acquisition of contextual fear response [17]. Our findings and earlier studies that produced contradictory findings [9, 10, 13–16, 30, 31] indicated that melatonin exerts facilitating or inhibiting effects on memory in different tasks. Taken overall, our present and previous results [17] suggest that melatonin exerts effects on the acquisition of contextual fear and cued fear extinction, but has no effect on cued fear conditioning. It is well established that cued fear conditioning is a hippocampus-independent task [32], while contextual fear conditioning requires the hippocampus [33–35]. Also, fear extinction is a form of active learning [20, 21]. Studies of the hippocampus have shown that the hippocampus is involved in the acquisition and consolidation of cued fear extinction, although the hippocampus is not essential for the acquisition of extinction per se [36, 37]. In the brain, binding sites of melatonin have been found in the amygdala, hippocampus and prefrontal cortex [38–40], three regions that are involved in fear extinction [21, 23]. Although we cannot be sure at which region melatonin acts to modulate extinction, these evidences suggest that the hippocampus may be the relevant sites.

Although the mechanisms through which melatonin acts on extinction remains to be determined, melatonin may have an indirect effect on memory formation via some neurotrophin such as brain derived neurotrophic factor (BDNF). Melatonin has been shown to increase the production of BDNF [41] that may play an important role in cued fear extinction [36]. Further, melatonin modulates neurotransmitters such as gama amino butyric acid (GABA) [42] and glutamate [43] which are involved in extinction learning [44]. Alternatively, the effects of melatonin may be through the direct modulation of memory formation circuits. Melatonin has shown to play an important role in structural remodeling of synaptic connections during memory and learning processes [45]. Other researches also demonstrated the ability of melatonin to modulate neuronal firing in the hippocampus and other brain regions [46, 47]. Further, a previous research showed that MT(2) receptor knockout mice demonstrates a significantly reduced long-term potentiation (LTP) as well as impaired memory performance tested in an elevated plus-maze paradigm [14]. Thus, melatonin may regulate learning and memory through its influence on synaptic connections in central nervous system neurons. Despite these evidences, the mechanism why Melatonin facilitates just extinction in our researches is still not known and there is at least one explanation. It was demonstrated that melatonin’s action on hippocampal LTP was mediated via the MT(2) melatonin receptor subtype through the regulation of adenylyl cyclase–protein kinase A (AC–PKA) pathway [47]. This pathway is critical for the formation of fear memory [48–50], but may be constraint for the extinction of fear because increased PKA activity impaired fear extinction [51, 52] whereas reduction of PKA activity facilitated fear extinction [50]. As melatonin inhibits activity of AC [53] and PKA [47], extinction of conditioned fear may be facilitated by melatonin through a mechanism involving regulation of the AC–PKA pathway. It is interesting to test this possibility in future research.

There has been an available effective means of extinction-based exposure psychotherapy for the treatment of anxiety disorders, such as PTSD [54, 55] which has been hypothesized to result from impaired extinction of fear memory [56, 57]. Furthermore, decreased melatonin levels in patients with PTSD were reported in clinical studies [58]. Therefore, facilitating effects of melatonin on fear extinction suggest that melatonin may serve as an agent for the treatment of anxiety disorders such as PTSD.

## Conclusion

In conclusion, the present observations demonstrate that intraperitoneal administration of melatonin (2.5 mg/kg) facilitated the extinction of conditional cued fear without affecting its acquisition or expression, and melatonin facilitated cued fear extinction only when it is present during extinction training. Additional studies will be required to specify the brain regions that mediate the extinction effects of melatonin as well as to explore mechanism through which melatonin exerts its effects on extinction learning.

## Abbreviations

ANOVA: Analysis of variance
CS: Conditioned stimulus
US: Unconditioned stimulus
i.p.: Intraperitoneally
LTP: Long-term potentiation
GABA: Gama amino butyric acid
BDNF: Brain derived neurotrophic factor
PTSD: Posttraumatic stress disorder.

## References

[1] Brzezinski A (1997). Melatonin in humans. N Engl J Med.

[2] Takahashi JS, Zatz M (1982). Regulation of circadian rhythmicity. Science.

[3] Cajochen C, Krauchi K, Wirz-Justice A (2003). Role of melatonin in the regulation of human circadian rhythms and sleep. J Neuroendocrinol.

[4] Barden N, Shink E, Labbe M, Vacher R, Rochford J, Mocaer E (2005). Antidepressant action of agomelatine (S 20098) in a transgenic mouse model. Prog Neuropsychopharmacol Biol Psychiatry.

[5] Mantovani M, Pertile R, Calixto JB, Santos AR, Rodrigues AL (2003). Melatonin exerts an antidepressant-like effect in the tail suspension test in mice: evidence for involvement of N-methyl-D-aspartate receptors and the L-arginine-nitric oxide pathway. Neurosci Lett.

[6] El-Sherif Y, Tesoriero J, Hogan MV, Wieraszko A (2003). Melatonin regulates neuronal plasticity in the hippocampus. J Neurosci Res.

[7] Rawashdeh O, Maronde E (2012). The hormonal Zeitgeber melatonin: role as a circadian modulator in memory processing. Front Mol Neurosci.

[8] Morgan PJ, Barrett P, Howell HE, Helliwell R (1994). Melatonin receptors: localization, molecular pharmacology and physiological significance. Neurochem Int.

[9] He P, Ouyang X, Zhou S, Yin W, Tang C, Laudon M, Tian S (2013). A novel melatonin agonist Neu-P11 facilitates memory performance and improves cognitive impairment in a rat model of Alzheimer' disease. Horm Behav.

[10] Bertaina-Anglade V, Drieu-La-Rochelle C, Mocaer E, Seguin L (2011). Memory facilitating effects of agomelatine in the novel object recognition memory paradigm in the rat. Pharmacol Biochem Behav.

[11] Argyriou A, Prast H, Philippu A (1998). Melatonin facilitates short-term memory. Eur J Pharmacol.

[12] Gorfine T, Zisapel N (2007). Melatonin and the human hippocampus, a time dependent interplay. J Pineal Res.

[13] Rimmele U, Spillmann M, Bartschi C, Wolf OT, Weber CS, Ehlert U, Wirtz PH (2009). Melatonin improves memory acquisition under stress independent of stress hormone release. Psychopharmacol (Berl).

[14] Larson J, Jessen RE, Uz T, Arslan AD, Kurtuncu M, Imbesi M, Manev H (2006). Impaired hippocampal long-term potentiation in melatonin MT2 receptor-deficient mice. Neurosci Lett.

[15] Rawashdeh O, de Borsetti NH, Roman G, Cahill GM (2007). Melatonin suppresses nighttime memory formation in zebrafish. Science.

[16] Karakas A, Coskun H, Kaya A, Kucuk A, Gunduz B (2011). The effects of the intraamygdalar melatonin injections on the anxiety like behavior and the spatial memory performance in male Wistar rats. Behav Brain Res.

[17] Yang Z, Li C, Huang F (2013). Melatonin impaired acquisition but not expression of contextual fear in rats. Neurosci Lett.

[18] LeDoux JE (2000). Emotion circuits in the brain. Annu Rev Neurosci.

[19] Maren S (2001). Neurobiology of Pavlovian fear conditioning. Annu Rev Neurosci.

[20] Bouton ME (1993). Context, time, and memory retrieval in the interference paradigms of Pavlovian learning. Psychol Bull.

[21] Bouton ME, Westbrook RF, Corcoran KA, Maren S (2006). Contextual and temporal modulation of extinction: behavioral and biological mechanisms. Biol Psychiatry.

[22] Sotres-Bayon F, Bush DE, LeDoux JE (2007). Acquisition of fear extinction requires activation of NR2B-containing NMDA receptors in the lateral amygdala. Neuropsychopharmacol.

[23] Sotres-Bayon F, Bush DE, LeDoux JE (2004). Emotional perseveration: an update on prefrontal-amygdala interactions in fear extinction. Learn Mem.

[24] Maren S, Quirk GJ (2004). Neuronal signalling of fear memory. Nat Rev Neurosci.

[25] Cho JH, Deisseroth K, Bolshakov VY (2013). Synaptic encoding of fear extinction in mPFC-amygdala circuits. Neuron.

[26] Lattal KM, Radulovic J, Lukowiak K (2006). Extinction: [corrected] does it or doesn't it? The requirement of altered gene activity and new protein synthesis. Biol Psychiatry.

[27] Lai CS, Franke TF, Gan WB (2012). Opposite effects of fear conditioning and extinction on dendritic spine remodelling. Nature.

[28] Marsicano G, Wotjak CT, Azad SC, Bisogno T, Rammes G, Cascio MG, Hermann H, Tang J, Hofmann C, Zieglgansberger W, Di Marzo V, Lutz B (2002). The endogenous cannabinoid system controls extinction of aversive memories. Nature.

[29] Kovacs GL, Gajari I, Telegdy G, Lissak K (1974). Effect of melatonin and pinealectomy on avoidance and exploratory activity in the rat. Physiol Behav.

[30] Soto-Moyano R, Burgos H, Flores F, Valladares L, Sierralta W, Fernandez V, Perez H, Hernandez P, Hernandez A (2006). Melatonin administration impairs visuo-spatial performance and inhibits neocortical long-term potentiation in rats. Pharmacol Biochem Behav.

[31] Gorfine T, Yeshurun Y, Zisapel N (2007). Nap and melatonin-induced changes in hippocampal activation and their role in verbal memory consolidation. J Pineal Res.

[32] Phillips RG, LeDoux JE (1992). Differential contribution of amygdala and hippocampus to cued and contextual fear conditioning. Behav Neurosci.

[33] Wallenstein GV, Vago DR (2001). Intrahippocampal scopolamine impairs both acquisition and consolidation of contextual fear conditioning. Neurobiol Learn Mem.

[34] Kim JJ, Fanselow MS (1992). Modality-specific retrograde amnesia of fear. Science.

[35] Chaudhury D, Loh DH, Dragich JM, Hagopian A, Colwell CS (2008). Select cognitive deficits in vasoactive intestinal peptide deficient mice. BMC Neurosci.

[36] Corcoran KA, Desmond TJ, Frey KA, Maren S (2005). Hippocampal inactivation disrupts the acquisition and contextual encoding of fear extinction. J Neurosci.

[37] Heldt SA, Stanek L, Chhatwal JP, Ressler KJ (2007). Hippocampus-specific deletion of BDNF in adult mice impairs spatial memory and extinction of aversive memories. Mol Psychiatry.

[38] Ekmekcioglu C (2006). Melatonin receptors in humans: biological role and clinical relevance. Biomed Pharmacother.

[39] Savaskan E, Ayoub MA, Ravid R, Angeloni D, Fraschini F, Meier F, Eckert A, Muller-Spahn F, Jockers R (2005). Reduced hippocampal MT2 melatonin receptor expression in Alzheimer's disease. J Pineal Res.

[40] Uz T, Arslan AD, Kurtuncu M, Imbesi M, Akhisaroglu M, Dwivedi Y, Pandey GN, Manev H (2005). The regional and cellular expression profile of the melatonin receptor MT1 in the central dopaminergic system. Brain Res Mol Brain Res.

[41] Kong X, Li X, Cai Z, Yang N, Liu Y, Shu J, Pan L, Zuo P (2008). Melatonin regulates the viability and differentiation of rat midbrain neural stem cells. Cell Mol Neurobiol.

[42] Rosenstein RE, Cardinali DP (1986). Melatonin increases in vivo GABA accumulation in rat hypothalamus, cerebellum, cerebral cortex and pineal gland. Brain Res.

[43] Vimala PV, Bhutada PS, Patel FR (2014). Therapeutic potential of agomelatine in epilepsy and epileptic complications. Med Hypotheses.

[44] Davis M, Myers KM (2002). The role of glutamate and gamma-aminobutyric acid in fear extinction: clinical implications for exposure therapy. Biol Psychiatry.

[45] Baydas G, Nedzvetsky VS, Nerush PA, Kirichenko SV, Demchenko HM, Reiter RJ (2002). A novel role for melatonin: regulation of the expression of cell adhesion molecules in the rat hippocampus and cortex. Neurosci Lett.

[46] Baydas G, Ozveren F, Akdemir I, Tuzcu M, Yasar A (2005). Learning and memory deficits in rats induced by chronic thinner exposure are reversed by melatonin. J Pineal Res.

[47] Wang LM, Suthana NA, Chaudhury D, Weaver DR, Colwell CS (2005). Melatonin inhibits hippocampal long-term potentiation. Eur J Neurosci.

[48] Abel T, Lattal KM (2001). Molecular mechanisms of memory acquisition, consolidation and retrieval. Curr Opin Neurobiol.

[49] Schafe GE, Nadel NV, Sullivan GM, Harris A, LeDoux JE (1999). Memory consolidation for contextual and auditory fear conditioning is dependent on protein synthesis, PKA, and MAP kinase. Learn Mem.

[50] Isiegas C, Park A, Kandel ER, Abel T, Lattal KM (2006). Transgenic inhibition of neuronal protein kinase A activity facilitates fear extinction. J Neurosci.

[51] Wang H, Ferguson GD, Pineda VV, Cundiff PE, Storm DR (2004). Overexpression of type-1 adenylyl cyclase in mouse forebrain enhances recognition memory and LTP. Nat Neurosci.

[52] Monti B, Berteotti C, Contestabile A (2006). Subchronic rolipram delivery activates hippocampal CREB and Arc, enhances retention and slows down extinction of conditioned fear. Neuropsychopharmacology.

[53] Boutin JA, Audinot V, Ferry G, Delagrange P (2005). Molecular tools to study melatonin pathways and actions. Trends Pharmacol Sci.

[54] Rothbaum BO, Davis M (2003). Applying learning principles to the treatment of post-trauma reactions. Ann N Y Acad Sci.

[55] Bentz D, Michael T, de Quervain DJ, Wilhelm FH (2010). Enhancing exposure therapy for anxiety disorders with glucocorticoids: from basic mechanisms of emotional learning to clinical applications. J Anxiety Disord.

[56] Milad MR, Rauch SL, Pitman RK, Quirk GJ (2006). Fear extinction in rats: implications for human brain imaging and anxiety disorders. Biol Psychol.

[57] Bremner JD, Vermetten E, Schmahl C, Vaccarino V, Vythilingam M, Afzal N, Grillon C, Charney DS (2005). Positron emission tomographic imaging of neural correlates of a fear acquisition and extinction paradigm in women with childhood sexual-abuse-related post-traumatic stress disorder. Psychol Med.

[58] McFarlane AC, Barton CA, Briggs N, Kennaway DJ (2010). The relationship between urinary melatonin metabolite excretion and posttraumatic symptoms following traumatic injury. J Affect Disord.

